# Supplementary material for: Nonlinear magnetotransport shaped by Fermi surface topology and convexity
Source: Nat Commun. 2019 Mar 20;10:1290. doi: 10.1038/s41467-019-09208-8 (PMC6426858; doi:10.1038/s41467-019-09208-8)
Supplement: Supplementary file 1 — Supplementary Information [file 41467_2019_9208_MOESM1_ESM.pdf]

SUPPLEMENTARY INFORMATION

Nonlinear magnetotransport shaped by Fermi surface topology and convexity

Pan He[1†], Chuang-Han Hsu[2,3†], Shuyuan Shi[1,2†], Kaiming Cai[1], Junyong Wang[2,3], Qisheng Wang[1], Goki Eda[2,3], Hsin Lin[4], Vitor M. Pereira[2,3] and Hyunsoo Yang[1,2]*

[1]*Department of Electrical and Computer Engineering, and NUSNNI, National University of Singapore, 117576, Singapore*

[2]*Centre for Advanced 2D Materials, National University of Singapore, 117546, Singapore*

[3]*Department of Physics, National University of Singapore, 117542 Singapore*

[4]*Institute of Physics, Academia Sinica, Taipei, 11529, Taiwan*

[†]These authors contributed equally to this work. *e-mail: eleyang@nus.edu.sg

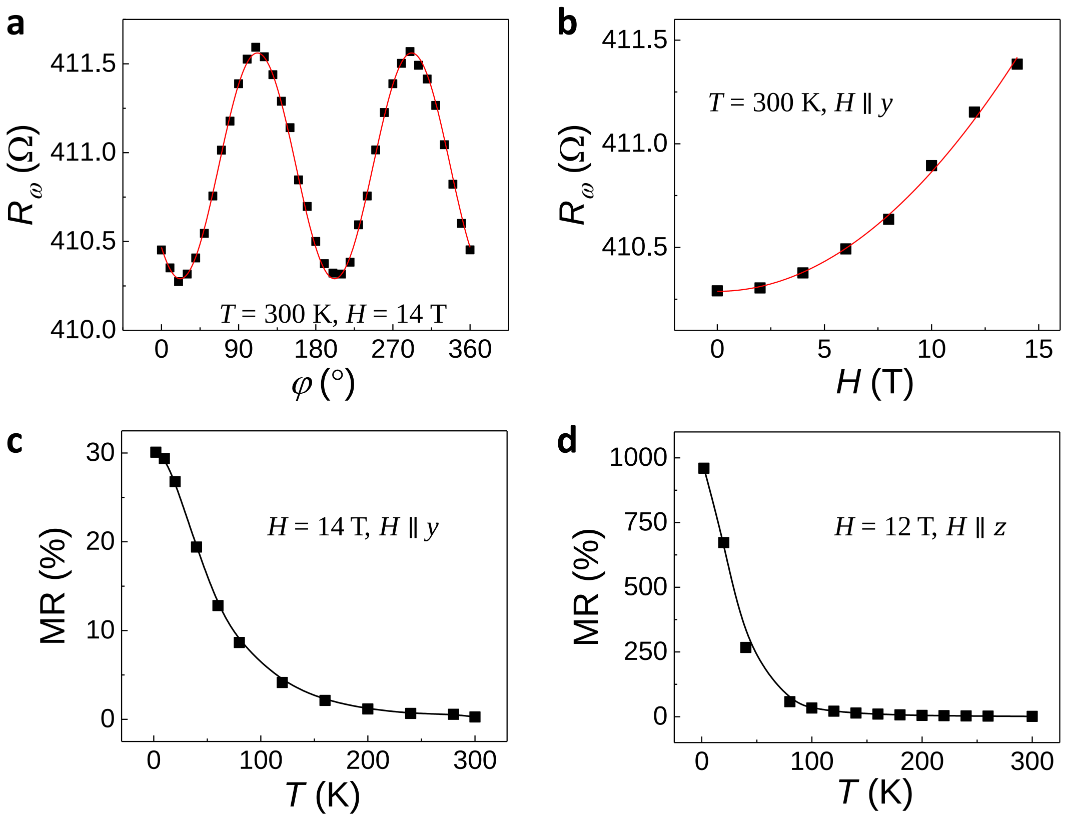

Supplementary Figure 1. Linear resistance R_ω and MR. **a**, R_ω vs. field-angle φ. R_ω shows an anisotropic magnetoresistance behavior with a period of 180°, revealing an invariant nature of the linear magnetoresistance under the reversal of magnetic field. **b**, R_ω vs. magnetic field H. R_ω shows a quadratic dependence with the magnetic field, in consistent with previous reports in WTe_2[1]. **c**,**d**, Temperature dependence of the linear MR measured at **H** ∥ y (**c**) and **H** ∥ z (**d**). The measurements were conducted at a 34 nm thick WTe_2 flake with the current applied at 45° titled from the a axis of WTe_2 crystal.

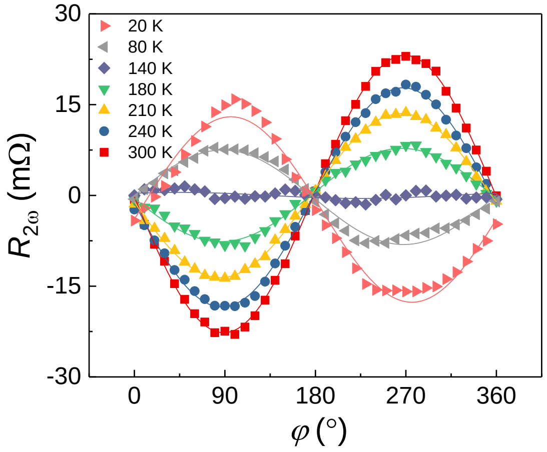

Supplementary Figure 2. Angular dependent $R_{2\omega}$ at different temperatures. A sign inversion of the $R_{2\omega}$ signal is found at T ~ 140 K. The measurements were conducted under H = 14 T and I = 1 mA for a 34 nm thick WTe_2 flake. The solid lines are sinusoidal fits to the data. A vertical offset in $R_{2\omega}$ was subtracted for clarity.

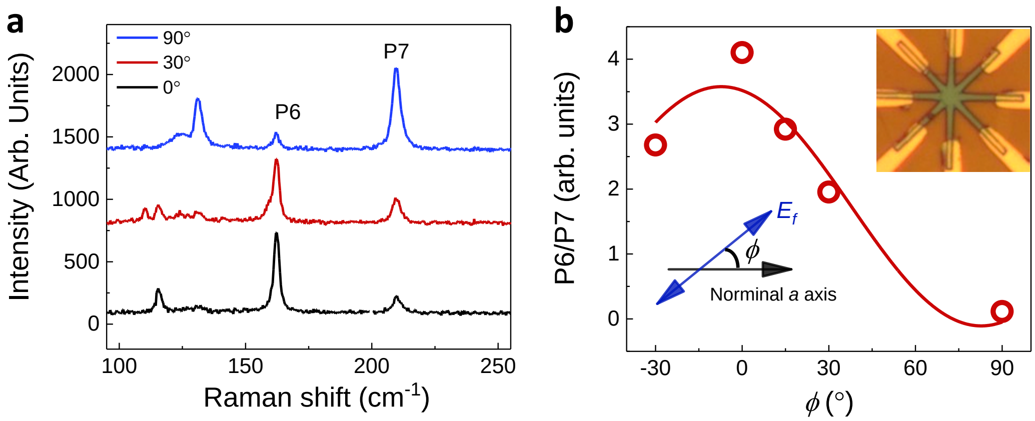

Supplementary Figure 3. Polarized Raman spectra. a, Polarized Raman spectra measured at different angle ϕ of electric field E_f directions of laser excitation with respect to the nominal *a* axis at room temperature. The peaks of P6 (~160 cm^{-1}) and P7 (~210 cm^{-1}) are marked, as defined in Ref. [2]. **b**, The peak intensity ratio of P6/P7 (red circle) as a function of ϕ. The *a* axis can be determined from the angle where the peak intensity of P6/P7 maximizes[3], which shows a small difference (< 8°) from the nominal one. The angle ϕ between E_f and the nominal *a* axis is defined in the inset of **b**.

Supplementary Note 1: Theoretical calculation of $J^{(2)}$

Under the relaxation time approximation, the single band Boltzmann equation in the presence of an electric field **E** is expressed as

$$-\frac{e\mathbf{E}}{\hbar}\cdot\frac{\partial f}{\partial \mathbf{k}} = -\frac{f-f_0}{\tau}, \quad (1)$$

where f_0 is the equilibrium Fermi-Dirac distribution and τ is the relaxation time (to be approximated as isotropic and constant). The solution for the distribution function f up to the second order in **E** is $f = f_0 + f_1 + f_2$ with $f_1 = \frac{-e\tau\mathbf{E}}{\hbar}\cdot\frac{\partial f_0}{\partial \mathbf{k}}$ and $f_2 = \frac{-e\tau\mathbf{E}}{\hbar}\cdot\frac{\partial f_1}{\partial \mathbf{k}} = \frac{e^2\tau^2\mathbf{E}}{\hbar^2}\cdot\frac{\partial^2 f_0}{\partial \mathbf{k}\partial \mathbf{k}}\cdot\mathbf{E}$. In our calculations, the quantity of interest is the second order longitudinal current density which, if the (linear) current flows along the x direction, is calculated as

$$J_{xx}^{(2)} = -\frac{e^3\tau^2E_x^2}{8\pi^2\hbar^2}\sum_n\int d\mathbf{k}^2 v_{x,n}(\mathbf{k})\frac{\partial^2 f_0}{\partial k_x^2}, \quad (2)$$

where $v_{x,n}(\mathbf{k})$ represents the velocity of the n-th band, and the contributions of each band are added independently. Since we are primarily interested in the variation of $J_{bb}^{(2)}$ with the temperature and magnetic field, and not in accurately capturing its magnitude, we leave the relaxation time unspecified and present all the results in an arbitrary scale.

From the density functional theory (DFT) band structure calculated for bulk WTe_2 (T_d phase), we build the best adapted tight-binding Hamiltonian $\mathcal{H}_{Bulk}(\mathbf{k})$ from maximally localized Wannier orbitals[4]. The band structure is shown in Fig. 1b, c. To incorporate the external magnetic field **H**, we add the Zeeman energy so that the total Hamiltonian reads $\mathcal{H}(\mathbf{k}) = \mathcal{H}_{Bulk}(\mathbf{k}) + \mu_B\mathbf{H}\cdot\boldsymbol{\sigma}$, where $\boldsymbol{\sigma}$ is the vector of Pauli matrices. The band velocities required for the numerical calculation of $J_{xx}^{(2)}$ according to Supplementary Equation 2 were obtained from the expectation value of $\langle v_x\rangle = \langle\frac{1}{\hbar}\frac{\partial\mathcal{H}(\mathbf{k})}{\partial k_x}\rangle$. The resulting longitudinal nonlinear charge current $J_{xx}^{(2)}$ along the b and a axes of WTe_2 under a field **H** in the plane but transverse to the current flow is shown in Supplementary Fig. 4 and 5, respectively. We find that the sign of $J_{bb}^{(2)}$ can be altered by changing either the temperature or Fermi

level μ. Though the sign inversion happens in $J_{aa}^{(2)}$ as well, the sign change is more prominent and robust for $J_{bb}^{(2)}$, in agreement with the experimental results shown in Fig. 3c and 3d. By separating the contributions to $J_{xx}^{(2)}$ arising from the hole and electron pockets, we find that the overall features of the nonlinear current are dominated by the electron pockets, especially the sign change for both current directions. This is shown in Supplementary Fig. 4b,c and Supplementary Fig. 5b,c.

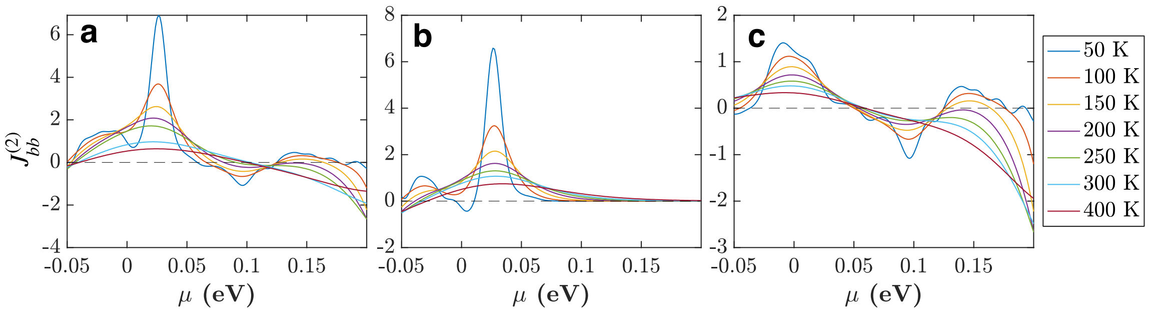

Supplementary Figure 4. a-c, Theoretically calculated second order charge current density $J_{bb}^{(2)}$ along the *b* direction for bulk WTe_2 taking into account (**a**) the entire pocket band structure, (**b**) the hole-pocket contributions, and (**c**) the electron-pocket contributions. A Zeeman energy of 0.1 meV was used.

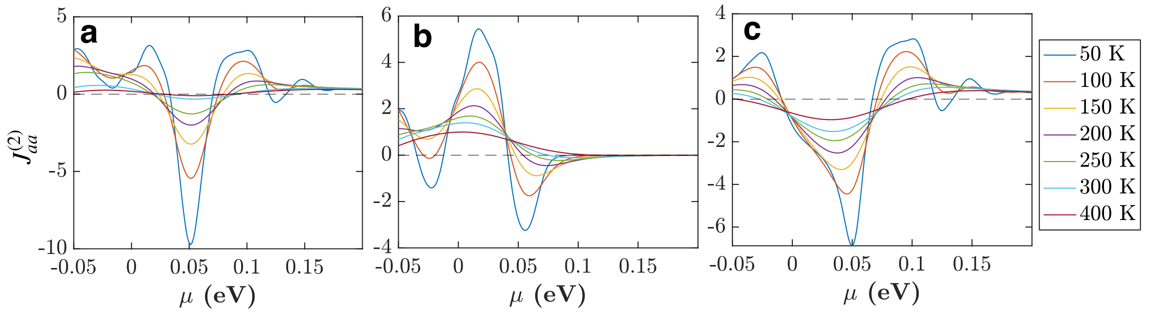

Supplementary Figure 5. a-c, Theoretically calculated second order charge current density $J_{aa}^{(2)}$ along the *a* direction for bulk WTe_2 taking into account (**a**) the entire pocket band structure, (**b**) the hole-pocket contributions, and (**c**) the electron-pocket contributions. A Zeeman energy of 0.1 meV was used.

Supplementary Note 2: Linear relation between $J^{(2)}$ and $R_{2\omega}$

As shown in Supplementary Note 1, under a magnetic field perpendicular to the electric field E_x in WTe_2, the longitudinal current density J_x includes a nonlinear term, $J_{xx}^{(2)}=\sigma_2 E_x^2$, in addition to the conventional linear one, $J_{xx}^{(1)}=\sigma_1 E_x$. The longitudinal current is therefore

$$J_x=\sigma_1 E_x+\sigma_2 E_x^2 \ . \tag{3}$$

Starting from the longitudinal resistivity,

$$\rho_{xx}=\frac{E_x}{J_x}=\frac{1}{\sigma_1+\sigma_2 E_x}\approx\frac{1}{\sigma_1}-\frac{\sigma_2 E_x}{\sigma_1^2}\,, \tag{4}$$

the longitudinal resistance R can be expressed as

$$R=R_0+R'(I)\,, \tag{5}$$

where $R_0=\frac{l}{wt\sigma_1}$ is the current-independent resistance, while $R'(I)=-\frac{\sigma_2}{wt\sigma_1^2}R_0 I$ is the current-dependent nonlinear resistance (l, w and t are the length, width and thickness of the Hall bar device). The nonlinear resistance $R'(I)$ can be measured by the harmonic technique, and the measured second harmonic resistance $R_{2\omega}$ is the half of the nonlinear resistance $R_{2\omega}=\frac{1}{2}R'(I)$.[5] Furthermore, from Supplementary Equation 4, $R_{2\omega}$ and $J_{xx}^{(2)}$ are linearly related as

$$R_{2\omega}=-\frac{lE_x}{2wt(J_{xx}^{(1)})^2}J_{xx}^{(2)}\,. \tag{6}$$

Supplementary Note 3: Temperature-induced Fermi level shift

The semimetal WTe_2 is known to have a Fermi level (μ) whose position changes markedly with temperature[6, 7], which in turn leads to large variations in the electron and hole densities. To quantify these density variations from a theoretical perspective, we compute the electron (n_e) and hole (n_h) densities as a function of μ predicted from our DFT-derived band structure.

The result, obtained with a 201×201×101 **k**-mesh, is shown in Supplementary Fig. 6. The zero Fermi energy ($\mu = 0$) is from DFT calculation, which is indicated by a dot-dashed line. It shows a similar trend as the experimentally extracted densities at the lowest measured temperature (2 K) in Fig. 2f, where the electron and hole carriers are nearly compensated. The experimental hole density drops by almost three orders of magnitude when the temperature increases from 2 to 300 K, and the magnitude of this reduction is captured by the calculated values in Supplementary Fig. 6 when μ varies between 0 and 120 meV. We note that this effective shift in μ (i.e. its rate of change with temperature) is in agreement with a previous APRES study[7] that find a temperature variation of 120 K induces a 50 meV variation in the Fermi level.

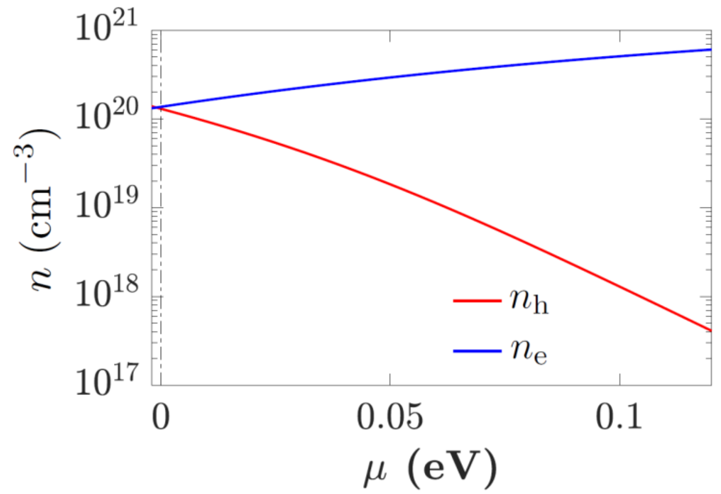

Supplementary Figure 6. Theoretically calculated electron (n_e) and hole (n_h) carrier densities versus the Fermi level μ for bulk WTe_2.

Supplementary Note 4: Quasi-bulk tight-binding model

In order to elucidate the detailed microscopic aspects underlying the sign inversion of $J_{xx}^{(2)}$ that we observed both experimentally (Fig. 2d) and theoretically (Fig. 4a and 4d), we develop a simpler quasi-bulk tight-binding model. Quasi-bulk means that a 2D tight-binding model is built by considering the symmetry properties of bulk WTe_2. It can be regarded as the effective 2D Hamiltonian at fixed k_z in the bulk[8]. We extend the $\mathbf{k}\cdot\mathbf{p}$ model that has been used to study monolayer 1T'-WTe_2[8], but consider an additional mirror symmetry and a term that breaks inversion symmetry to make it compatible with the T_d structure of WTe_2 in our experiments. The symmetry operators read

$$T = is_y \otimes I,\ \ \{M_x|0\} = is_x \otimes I,\ \ \{M_y|\tau\} = is_y \otimes \tau_x,\ \ P = I \otimes \tau_z,$$

where the Pauli matrices s and τ are associated with the spin and sublattice degrees of freedom, and $T, M,$ and P represent the time-reversal, mirror, and inversion symmetries. Considering these symmetries, the quasi-bulk $\mathbf{k}\cdot\mathbf{p}$ Hamiltonian becomes

$$\mathcal{H}(\vec{k}) = m_1 k^2 + (m_2 k^2 + \delta) I \otimes \tau_z + \alpha k_y s_x \otimes \tau_x + \beta k_y I \otimes \tau_y$$

$$+\gamma k_x s_y \otimes \tau_x + \eta I \otimes \tau_x,$$

where m_1 and m_2 control the effective mass of two effective bands, δ describes the strength of band inversion among two effective bands, and α and γ are spin-orbit couplings. β is the anisotropy term that changes the dispersion along k_y, and η in the last term is the inversion breaking strength.

Once this low energy $\mathbf{k}\cdot\mathbf{p}$ model is established, we continue it on a rectangular lattice to have a Hamiltonian defined over the entire Brillouin zone. This extension defines what we designate by quasi-bulk tight-binding model. Its associated band structure is shown in Supplementary Fig. 7a and, for a direct comparison, the DFT band structure of bulk WTe_2 at $k_z = 0$ and π is displayed in Supplementary Fig.7b and 7c, respectively. This simplified model captures qualitatively well the key characteristics of the bands near the Fermi energy, especially when it comes to the electron pockets which, as we mentioned in Supplementary Note 1, are the main contributors to the inversion of the nonlinear current. Most importantly, the concave and convex shapes of the Fermi surfaces are well reproduced by this model when varying the Fermi level, as detailed in the main text (Fig. 4b,c and Fig. 4h,i).

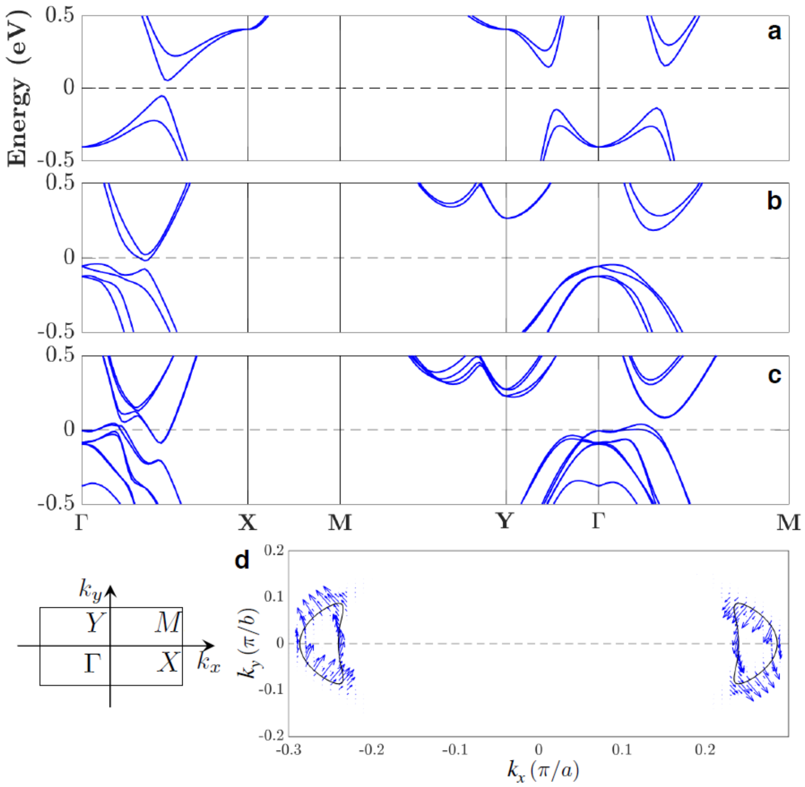

Supplementary Figure 7. a-c, Band structure based on (**a**) the quasi-bulk tight-binding model. Band structure based on DFT at (**b**) $k_z = 0$ and (**c**) $k_z = \pi$ in bulk WTe_2. **d**, The Fermi surface and in-plane spin texture calculated from the quasi-bulk tight-binding model.

Supplementary Note 5: J (E^2) in a spin polarized parabolic band

Adding the Zeeman coupling to the **k · p** model discussed in Supplementary Note 4, and solving for the energy dispersion, the lowest spin-polarized conduction band has the following momentum dependence,

$$E(k_y, H) = m_y k_y^2 + \xi_1 H_x k_y + \xi_2 H_x k_y^3 \tag{7}$$

where m_y is an effective mass that depends on k_x, and ξ_1 and ξ_2 are factors arising from the Zeeman energy. x and y identify the a and b axes of T_d-WTe_2, respectively. Using this band dispersion to compute the nonlinear current according to Supplementary Equation 2, we obtain

$$J_{yy}{}^{(2)} \sim -\left(18 m_y \xi_2 H_x |\delta| + \frac{9\xi_1^2 \xi_2 H_x^3}{2m_y}\right). \tag{8}$$

This result shows that the sign of $J_{yy}^{(2)}$ is governed by that of m_y. As discussed in the main text, an inversion of the sign of m_y translates into Fermi contours which are not globally convex.

In Supplementary Fig. 8a and 8b, we show this aspect in more detail by inspecting the k-resolved $J_{yy}^{(2)}(k_x, k_y)$ at the two representative energies indicated by the dashed vertical lines in Fig. 4g of the main text. Integrating $J_{yy}^{(2)}(k_x, k_y)$ over k_y, we can extract the overall contributions to the nonlinear current at each k_x, which are shown in Supplementary Fig. 8c and 8d. The mostly positive values shown in Supplementary Fig. 8c can be interpreted directly from Supplementary Equation 8, because the energy dispersion is globally parabolic with a convex Fermi contour. On the other hand, the case shown in Supplementary Fig. 8d exhibits a number of sign changes. However, it is clear that the strongest negative contribution arises from the portions of the Brillouin zone where the Fermi surface displays a concave shape. This contribution dominates and determines the final negative sign upon integration over k_x to recover $J_{yy}^{(2)}$.

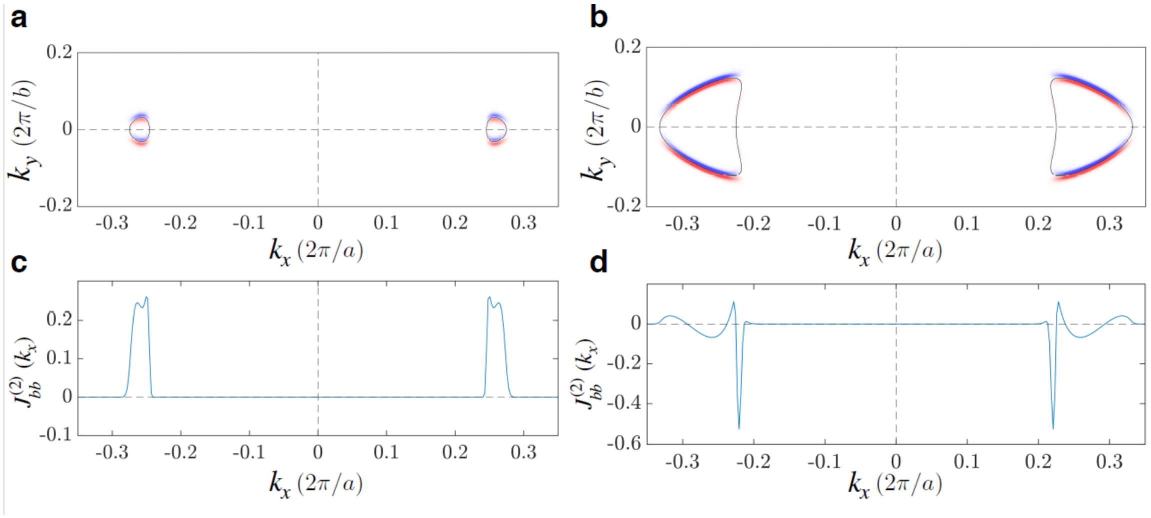

Supplementary Figure 8. a,**b**, *k*-space-resolved $J^{(2)}_{bb}(k_x, k_y)$ at (**a**) E = 0.075 eV and (**b**) E = 0.2 eV computed with the quasi-bulk tight-binding model. Red and blue color represent positive and negative values, and the black curves are the Fermi contours. **c**,**d**, The *k*-resolved current density integrated over k_y at the energies considered in panels **a** and **b**, respectively.

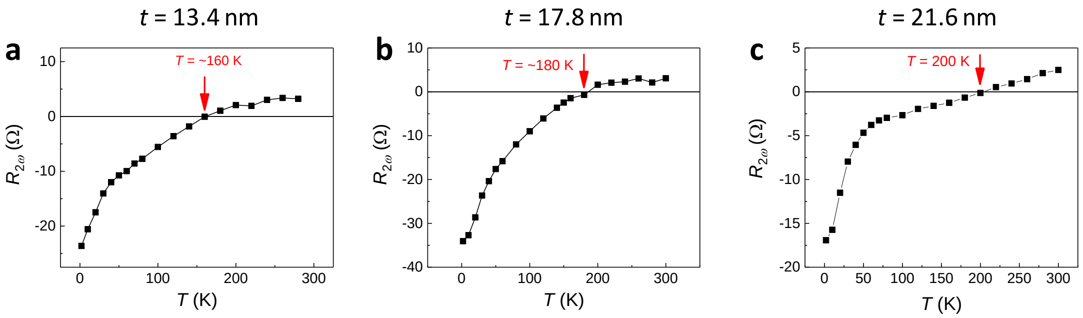

Supplementary Figure 9. Sign inversion temperature of $R_{2\omega}$ for different flake thicknesses. a-c, $R_{2\omega}$ (normalized at H = 1 T and I = 1 A) as a function of temperature for the WTe_2 flake thickness of t = 13.4 nm (**a**), 17.8 nm (**b**), and 21.6 nm (**c**), with the current applied along the *b* axis.

Supplementary References

1. Ali, M. N. et al. Large, non-saturating magnetoresistance in WTe_2. *Nature* **514,** 205 (2014).
2. Kong, W.-D. et al. Raman scattering investigation of large positive magnetoresistance material WTe_2. *Appl. Phys. Lett.* **106,** 081906 (2015).
3. MacNeill, D. et al. Control of spin–orbit torques through crystal symmetry in WTe_2/ferromagnet bilayers. *Nat. Phys.* **13,** 300 (2016).
4. Chang, T.-R. et al. Prediction of an arc-tunable Weyl Fermion metallic state in $Mo_xW_{1-x}Te_2$. *Nat. Commun.* **7,** 10639 (2016).
5. He, P. et al. Observation of out-of-plane spin texture in a $SrTiO_3$(111) two-dimensional electron gas. *Phys. Rev. Lett.* **120,** 266802 (2018).
6. Luo, Y. et al. Hall effect in the extremely large magnetoresistance semimetal WTe_2. *Appl. Phys. Lett.* **107,** 182411 (2015).
7. Wu, Y. et al. Temperature-induced Lifshitz transition in WTe_2. *Phys. Rev. Lett.* **115,** 166602 (2015).
8. Qian, X., Liu, J., Fu, L. & Li, J. Quantum spin Hall effect in two-dimensional transition metal dichalcogenides. *Science* **346,** 1344-1347 (2014).
